# Integrative Taxonomy Does Not Support a Cosmopolitan *Desmophyllum pertusum*: Resurrecting *D. californicum* and Validating the Genus *Desmophyllum*

**DOI:** 10.1002/ece3.74485

**Published:** 2026-10-16

**Authors:** Claudia F. Vaga, Alexis M. Weinnig, Stephen D. Cairns, Michelle L. Taylor, Meredith Everett, Santiago Herrera, Erik E. Cordes, Andrea M. Quattrini, Cheryl L. Morrison

**Affiliations:** ^1^ Department of Invertebrate Zoology National Museum of Natural History, Smithsonian Institution Washington DC USA; ^2^ Eastern Ecological Science Center, Leetown Research Laboratory U.S. Geological Survey Kearneysville West Virginia USA; ^3^ University of Essex Oxford UK; ^4^ NOAA Northwest Fisheries Science Center Seattle Washington USA; ^5^ Department of Biological Sciences Lehigh University Bethlehem Pennsylvania USA; ^6^ Oceans Research Center Lehigh University Bethlehem Pennsylvania USA; ^7^ Department of Biology Temple University Philadelphia Pennsylvania USA

**Keywords:** conservation, genetic diversity, *Lophelia*, Scleractinia, systematics

## Abstract

Reef‐building corals are key contributors in marine ecosystems, from warm, shallow reefs to cold, deep‐water habitats worldwide. *Desmophyllym pertusum* (Linnaeus, 1758) is one of the most important habitat‐forming cold‐water corals and has long been considered a cosmopolitan species. Yet phylogenetic relationships within the genus, as well as its relatedness to the synonymized genus *Lophelia* (Milne Edwards & Haime, 1849), remain poorly understood and debated. Here, we use nuclear ultraconserved elements and exons, complete mitochondrial genomes, and single nucleotide polymorphism data to show that populations of *D. pertusum* in the eastern and central North Pacific Ocean are genetically differentiated from those in the western and eastern Atlantic Ocean. Our results support the previously proposed synonymy of genera *Lophelia* and *Desmophyllum* (Ehrenberg, 1834). Morphological features of specimens from the Pacific Ocean, particularly budding pattern and corallite size, match those of the previously synonymized *D. californicum* comb. nov., which we therefore reinstate. We also incorporated solitary *Desmophyllum* spp. lineages into phylogenomic analyses, including a multispecies coalescence model. Taken together, these integrative taxonomic results indicate the presence of multiple *Desmophyllum* species beyond those currently recognized as valid. Future studies could incorporate phylogenomic approaches to better delimit species boundaries and avoid grouping distinct coral species.

## Graphical abstract

Reef‐building cold‐water corals are important habitat‐forming species, yet the taxonomy of the widely distributed *Desmophyllum pertusum* remains uncertain. Using an integrative approach combining nuclear UCEs and exons, mitochondrial genomes, SNP data, and morphology, we show that Pacific populations previously identified as *D. pertusum* are genetically and morphologically distinct from Atlantic populations, supporting the resurrection of *Desmophyllum californicum*. Our results also support the synonymy of *Lophelia* and *Desmophyllum* and highlight previously unrecognized diversity within this ecologically important coral genus.

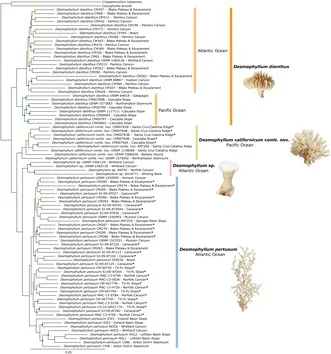

## 1 Introduction

Cold‐water corals (CWCs) are a key contributor to marine ecosystems, particularly in deep‐sea environments (S. D. Cairns 2007). Many CWCs form microhabitats by constructing complex three‐dimensional structures that provide shelter and resources for a wide range of micro‐ and macroorganisms (Buhl‐Mortensen et al. 2010). These habitats support biodiversity and play a central role in organic carbon cycling, underscoring their ecological importance in the deep sea (e.g., Cathalot et al. 2015).

Scleractinian (Anthozoa, Hexacorallia) CWCs can exist as solitary individuals or form large colonial structures. In colonial species, hundreds to thousands of polyps may be housed within corallites and arranged in an arborescent framework, functioning collectively as a single organism (Freiwald and Wilson 1998). The most extensively studied CWC is the colonial scleractinian *Desmophyllum pertusum* (Linnaeus, 1758) (= *Lophelia pertusa* (Pallas, 1766)), which accounts for approximately 30% of all published research on scleractinian CWCs (Vuleta et al. 2024).

*Desmophyllum pertusum* is a dominant azooxanthellate habitat‐forming species (family Caryophylliidae) with a nearly global distribution (S. D. Cairns 1994). This species is bathymetrically widespread (found from 40 to 2775 m), inhabits a variety of habitat types (e.g., seamounts, submarine canyons, and rocky outcroppings), creates bioherms and habitat for other organisms, and exhibits high phenotypic plasticity in colony growth forms in response to varying environmental conditions (Mortensen et al. 1995; Reed et al. 2006; Sanna and Freiwald 2021). Despite the ecological importance and cosmopolitan occurrence of *D. pertusum* , comprehensive studies of its population connectivity and genetic diversity have been lacking worldwide, thus hampering correct identification and conservation efforts. Indeed, relying solely on morphological observations, several nominal species described from different ocean basins have been synonymized with *D. pertusum* over the past few decades (e.g., S. D. Cairns 1979, 2000).

Previous population genetic studies have revealed substantial geographic structure in *D. pertusum* across the western North Atlantic Ocean. Microsatellite analyses showed strong differentiation among populations in the North Atlantic Ocean (Morrison et al. 2011; Lunden et al. 2014). More recently, SNP analyses revealed high connectivity among populations along the eastern U.S. continental margin, which were genetically differentiated from populations in Norfolk Canyon and in the Gulf of America (formerly Gulf of Mexico). Interestingly, despite their geographic separation, Norfolk Canyon and Gulf populations showed low genetic differentiation (Weinnig et al. 2024). At a broader phylogenetic scale, mitochondrial and nuclear data revealed extremely low genetic divergence between *D. pertusum* and *D. dianthus* (Esper, 1794) (Addamo et al. 2016; Seiblitz et al. 2022).

The synonymy of the genus *Lophelia* (Milne Edwards & Haime, 1849) with *Desmophyllum* (Ehrenberg, 1834) was recently proposed based on data from mitochondrial genomes, 30 microsatellite markers, and microscale skeletal characteristics (Addamo et al. 2016, but also see Zibrowius 1980). Given the extensive research conducted under the name *L. pertusa* (summarized in Vuleta et al. 2024) and the notable morphological differences between solitary (*Desmophyllum*) and colonial (*Lophelia*) forms (e.g., S. D. Cairns 1994), this taxonomic revision was initially contentious in the field. Indeed, coloniality, or the lack of it, has been historically used as an informative taxonomic character at the genus level for scleractinian corals (e.g., Cairns and Kitahara 2012). However, in the past few years, systematic studies using integrative taxonomy have confirmed that some genera include both solitary and colonial species (Hoeksema 2012; Mehrotra et al. 2023). The synonymy of the two genera was ultimately accepted by the World Register of Marine Species (WoRMS) editors (Hoeksema and Cairns 2025). Despite this acceptance, some researchers have suggested that more comprehensive molecular evidence is needed to support the validity of the reclassification (Cairns, note in WoRMS).

Genomic analyses are rapidly advancing scleractinian systematics. Integrating population genomics, nuclear phylogenomics using ultraconserved elements (UCEs) and exon loci, and mitochondrial genomics provides a powerful, multilocus framework for resolving species boundaries with high precision within corals (e.g., Erickson et al. 2021; Bridge et al. 2024; Terraneo et al. 2025). When integrated, these methods allow researchers to assess congruence between nuclear and mitochondrial signals, detect cryptic speciation, and resolve taxonomic uncertainties with greater confidence, especially in groups with complex evolutionary histories or limited morphological differentiation (e.g., Capel et al. 2024). Genomic data combined with morphological data are necessary to support synonymy of the genera *Lophelia* and *Desmophyllum* and validity of recognized species.

In this study, we use an integrative taxonomic approach to clarify the evolutionary relationships and the taxonomic validity of the genus *Desmophyllum*. Specifically, we combine population genomic, nuclear phylogenomic, and mitochondrial genomic data to assess whether the synonymy of the genus *Lophelia* with *Desmophyllym* is supported by genome‐scale evidence. Furthermore, we propose the resurrection of the species *Lophelia californica* Durham 1947 under the new combination *Desmophyllum californicum* to designate a genetically and morphologically distinct species found along the west coast of North America, from Baja California to Canada, and Hawaii.

## 2 Materials and Methods

### 2.1 Methods Overview and Rationale

For this study, specimens of *D. pertusum* and *D. dianthus* were collected from multiple locations in both the Atlantic and Pacific Oceans to assess biodiversity, genetic differentiation, and the genus validity. The genus *Desmophyllum* currently includes only four species: *D. dianthus* , *D. hourigani* Cairns, 2021, *D. pertusum* , and *D. quinarium* Tenison‐Woods, 1879. *Desmophyllum quinarium* has not been collected since its original description, and no specimens of *D. hourigani* suitable for DNA analyses are currently available. Consequently, only specimens of the widespread *D. pertusum* and *D. dianthus* could be included in the analyses. We initially analyzed *D. pertusum* specimens from the east and west coasts of the United States (U.S.) to evaluate population structure across these geographically distant populations through RADSeq sequencing data including the dataset generated by Weinnig et al. (2024). Given that population connectivity across locations along the U.S. east coast had already been investigated (Lunden et al. 2014; Weinnig et al. 2024), our analyses focused on genetic differentiation between the Pacific and Atlantic Ocean basins.

Preliminary results revealed a clear genetic separation between the two regions. To explore these patterns further, we sequenced *D. pertusum* and *D. dianthus* specimens through low‐coverage whole‐genome sequencing (herein referred as ‘genome skimming’) methods to recover UCEs and complete mitochondrial genomes. The dataset was further expanded with historical samples of both species sourced from the Smithsonian National Museum of Natural History ([NMNH]; otherwise known as the U.S. National Museum [USNM]). Using this dataset, species relationships were assessed through phylogenetic reconstructions (maximum likelihood and species tree analyses). The two datasets represent complementary stages of a multi‐year study and provide partially independent lines of evidence. Complete mitochondrial genomes were retrieved from the genome skimming sequencing data for a subset of *D. pertusum* specimens to evaluate within and between species differences and compare the results to prior mitochondrial studies on the genus *Desmophyllum* (Addamo et al. 2016; Seiblitz et al. 2022). Specimens of *D. pertusum* collected from the Pacific Ocean were confirmed to represent a distinct species, leading to the resurrection of *Desmophyllum californicum* comb. nov. (see Taxonomic Account below). For clarity, all Pacific Ocean specimens previously identified as *D. pertusum* are hereafter referred to as *D. californicum* comb. nov.

### 2.2 Sample Collections

#### 2.2.1 Contemporary Collections

Contemporary samples of *D. californicum* comb. nov., *D. dianthus* , and *D. pertusum* were collected over a span of ten years from the north Atlantic Ocean and the eastern north Pacific Ocean. Sampling localities can be found in Table S1 and Figure 1. We also indicate location designations reflecting *D. pertusum* population structure patterns reported in previous studies (Morrison et al. 2011; Lunden et al. 2014; Weinnig et al. 2024). All samples were collected by submersible vehicles including human‐occupied vehicles *Johnson Sea‐Link* and *Alvin* and remotely operated vehicles (ROVs) *SuBastian, Jason*, and *Hercules*. Upon collection, the samples were either preserved onboard the vessel for genetic analysis (95% EtOH or flash frozen in liquid nitrogen) or kept alive and brought back to shore, later preserved for genetic analysis.

**FIGURE 1:**
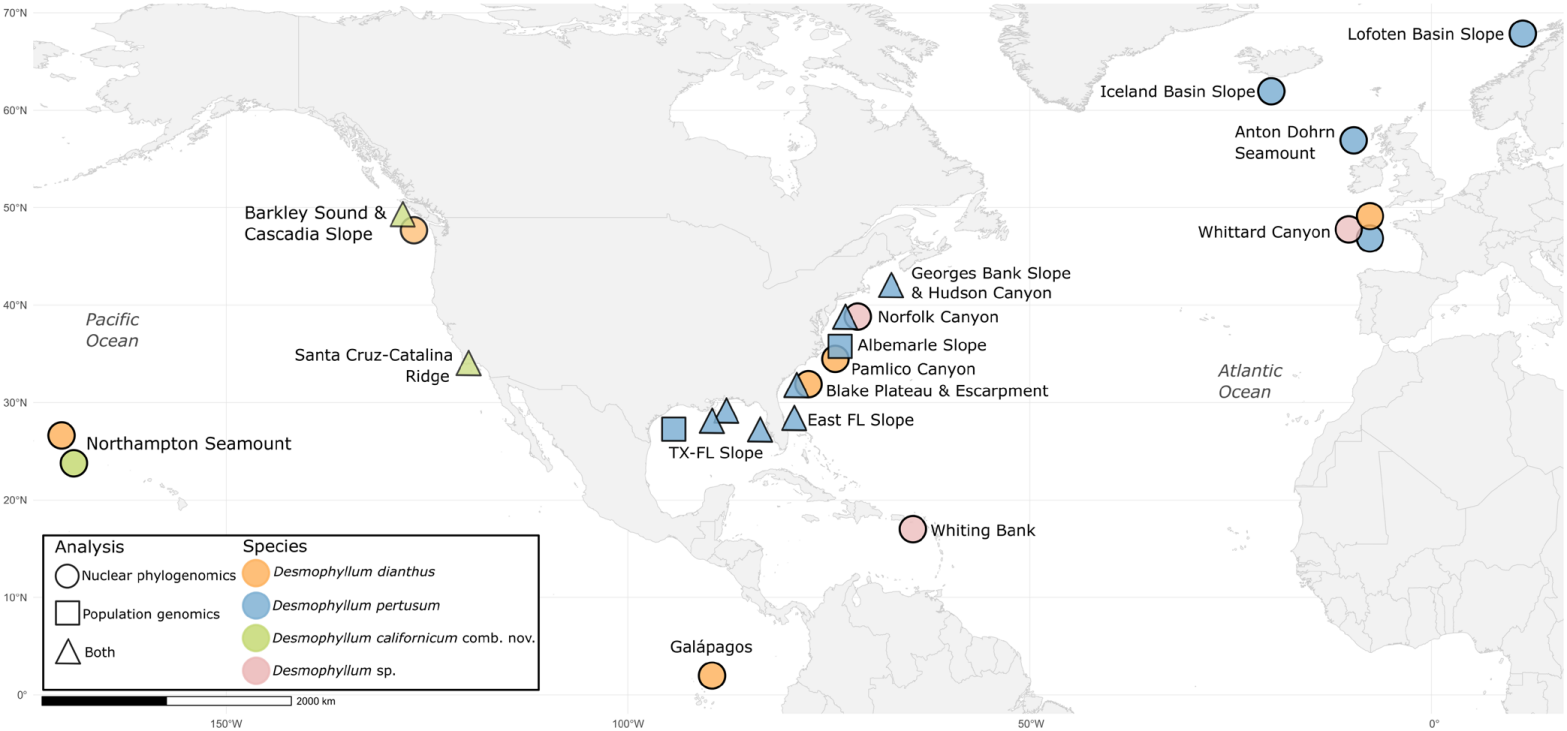
Map of geographic regions where samples were collected (locations shown are approximate) and whether they were used for nuclear phylogenomics or population genomics. Species listed are according to the maximum likelihood phylogeny shown in Figure 5. TX = Texas, FL = Florida. Base map derived from Natural Earth data through the R package maps (Becker et al. 2025).

In the central and eastern north Atlantic Ocean basin, specimens were collected at sites from the Iceland Basin Slope (*n* = 2— *D. pertusum* ; hosted at the Marine and Freshwater Research Institute, Iceland, care of Dr. Steinunn Hilma Ólafsdóttir), Anton Dohrn Seamount (*n* = 2— *D. pertusum* ; hosted at the University of Essex, England, care of Dr. Michelle Taylor), Whittard Canyon (*n* = 2— *D. pertusum* ; hosted at the National Oceanography Centre care of Tammy Horton), and the Lofoten Basin Slope (*n* = 2— *D. pertusum* ; hosted at the Institute of Marine Research, Norway, care of Dr. Tina Kutti). In the western north Atlantic Ocean, samples were collected from the southeast slope of Georges Bank between Munson and Kinlan Canyons (collectively referenced herein as the Georges Bank Slope, *n* = 8— *D. pertusum* ), Norfolk Canyon (*n* = 11— *D. pertusum* ), and Pamlico Canyon (*n* = 10— *D. dianthus* ). We included one sample from a chemosynthetic, cold seep located on the east slope of the Albemarle Shelf Valley five miles north of Keller Canyon and 37 miles east of Pea Island, North Carolina, USA (referenced herein as the Albemarle Slope and by Cordes et al. (2024) and Weinnig et al. (2024) as “Pea Island” *n* = 1— *D. pertusum* ). Samples were collected from sites across the Blake Plateau and Escarpment, where populations are highly connected (*n* = 33— *D. pertusum* , *n* = 9— *D. dianthus* ). Along the southern coast of the USA, samples were collected from the continental slope between Texas (TX) and Florida (FL) (specifically, from the west Florida Slope west of Fort Myers, FL, at Viosca Knolls, and at locations south of East Flower Garden Bank and south of Barataria Bay on the Texas–Louisiana Slope, collectively referenced as TX‐FL Slope *n* = 25— *D. pertusum* ), and from the Florida‐Hatteras Slope east of Cape Canaveral (referenced herein as the east FL Slope and by Cordes et al. (2024) as “Canaveral” *n* = 18— *D. pertusum* ), Florida, USA. In the Caribbean Sea, samples were collected from Whiting Bank, south of Puerto Rico (*n* = 1— *D. dianthus* ). In the Pacific Ocean, we collected samples along the Santa Cruz‐Catalina Ridge west of Los Angeles, California, USA (*n* = 3— *D. californicum* comb. nov.), and from the Juan de Fuca and Quillayute Canyons on the Cascadia Slope west of the Olympic Peninsula, Washington, USA (referenced herein as “Cascadia Slope” *n* = 2— *D. californicum* comb. nov., *n* = 5— *D. dianthus* ), collectively referenced herein as North Pacific, *n* = 10. Where not previously specified, voucher specimens were either depleted during molecular analyses or deposited in the collections of the Smithsonian NMNH (see Table S1 for NMNH voucher numbers).

#### 2.2.2 Museum Collections

Historical specimens preserved in either 70% or 95% ethanol of *D. californicum* comb. nov., *D. dianthus* , and *D. pertusum* (*n* = 13, Table S1) were sourced from the NMNH collection (cataloged with USNM numbers). We chose specimens that included a range of geographic diversities from similar sites (i.e., Santa Cruz‐Catalina Ridge, California [*n* = 1— *D. californicum* comb. nov.], Juan de Fuca Canyon [*n* = 1— *D. dianthus* ], Munson Canyon [*n* = 3— *D. pertusum* ], Whittard Canyon [*n* = 3— *D. dianthus* ]) and not covered by the contemporary collections (i.e., Hudson Canyon, due east of Atlantic City, New Jersey, USA [*n* = 1— *D. dianthus* ]; Barkley Sound on the southwest margin of Vancouver Island, British Columbia, Canada [*n* = 1— *D. californicum* comb. nov.*m*]; Northampton Seamount, Hawaii, USA, [*n* = 1— *D. californicum* comb. nov., *n* = 1— *D. dianthus* ]; and ~28 km SSE of Marchena Island, Galapagos, Ecuador [*n* = 1— *D. dianthus* ]).

### 2.3 DNA Extraction and Sequencing Methods

#### 2.3.1 RADSeq Sequencing

DNA was extracted from 96 *D. pertusum* and 5 *D. californicum* comb. nov. samples, but the age and quality of tissue varied; therefore, several DNA extraction methods were used to obtain the quality and quantity of high molecular weight DNA required for sequencing. Multiple extraction kits including the Qiagen PureGene, Qiagen DNeasy Blood & Tissue Kit, and a modified CTAB method (France et al. 1996) were employed across the sample set, depending on tissue quality. The Qubit dsDNA BR Assay Kit (Thermo Fisher) was used to quantify the DNA from each sample, and the quality was assessed through 2.0% agarose gel electrophoresis. The DNA was then cleaned using either the Qiagen DNeasy PowerClean Pro Cleanup Kit or Ampure magnetic beads (Agencourt Bioscience Corporation). Next, each sample was run through a test enzyme digestion using the *mseI* restriction enzyme (New England Bio) to ensure enzymatic inhibitors were removed during the cleaning steps. Following the DNA extraction and cleaning methods, DNA was normalized to 20 ng/μL and sent for restriction‐site associated DNA sequencing (RADSeq) library preparation and sequencing. The DNA libraries were constructed using the 6‐cutter *PstI* and *Mse1* enzymes and sequenced on an Illumina HiSeq4000 (100‐bp single‐end reads) at University of Oregon's Genomics and Cell Characterization Core Facility lab.

#### 2.3.2 Genome Skimming Sequencing

Total genomic DNA extraction of specimens selected for phylogenomic analyses (see Table S1 and Methods Section 2.1) was performed using the Qiagen DNeasy Blood and Tissue kit following the manufacturer's animal tissue protocol. DNA purification was then performed using the Genomic DNA Clean & Concentrator kit (Zymo Research). DNA integrity was assessed through 1.5% agarose gel electrophoresis. DNA was sheared to a target size range of 400–800 bp using sonication (Q800R QSonica sonicator), and library preparation was conducted with a NEBNext Ultra II DNA Library Prep Kit with some modifications (Morrissey et al. 2026): the reaction volume was reduced by half, iTru Y‐yoke oligonucleotide adapters (Glenn et al. 2019) were used instead of NEBNext Adaptors, adaptor ligation time was 30 min, iTru i5 and i7 indices (Glenn et al. 2019) were used, and 13 cycles of PCR enrichment were conducted. DNA concentration before and after library preparation was quantified with a Qubit dsDNA HS Assay Kit (Thermo Fisher), and size distributions after library preparation were assessed on a TapeStation (Agilent Technologies). Samples were sequenced through genome skimming on an Illumina NovaSeq 6000 (150‐bp paired‐end reads) at the Oklahoma Medical Research Foundation Genomics Core.

### 2.4 Sequence Processing and Bioinformatics

#### 2.4.1 Population Genomics

The RADSeq data were demultiplexed and assembled using default parameters in ipyrad v 0.9.81 (Eaton and Overcast 2020) under *reference* mode, using the published *D. pertusum* (= *Lophelia pertusa* ) genome as reference (Herrera and Cordes 2023). Loci and single nucleotide polymorphism (SNP) information were recovered in variant call format (vcf) and then filtered using vcftools v 0.1.16 (Danecek et al. 2011). Using vcftools, the thin function was used to retain one SNP per locus to obtain unlinked SNPs. The ‐‐missing‐indv function calculated the percent of missing data of each individual. Individuals with more than 85% missing data were removed (‐remove) from the dataset. Further filtering was applied to keep only the variants that successfully genotyped at least 50% of individuals (‐max‐missing 0.5) and a minimum allele count of 3 (‐mac 3).

Individuals analyzed for population genomics were assigned to six populations as noted above in Section 2.2.1: Georges Bank Slope, Norfolk Canyon, Blake Plateau and Escarpment, east FL Slope, TX‐FL Slope, and North Pacific (see Table S1). Additionally, the one sample collected from the Albemarle Slope is included in principal component analysis (PCA) and STRUCTURE v 2.3.4 (Pritchard et al. 2000). The API: ipyrad‐analysis‐toolkit was used to run and plot the PCA and STRUCTURE analysis. Within the toolkit, each population was required to maintain a minimum of 25% of the samples, and SNPs that were shared across less than 10% of all of the samples were excluded. STRUCTURE was run in triplicate (*n* = 3, burnin = 20,000, numreps = 100,000) using population (K) values up to more than one more than the number of populations (K = 2–7). Using the R (v 4.1.1) (R Core Team 2021) packages *adgenet* and *hierfstat*, pairwise *F*_ST_ values were calculated between assigned populations according to Weir and Cockerham (1984).

#### 2.4.2 Phylogenomic Analyses

Quality control and adapter removal of demultiplexed sequencing data were performed with Trimmomatic v 0.39 (Bolger et al. 2014), and trimmed sequences were assembled into contigs using SPAdes v 3.15 (Bankevich et al. 2012). At this stage, previously published genomic data of *Desmophyllum* species and two other caryophylliid species ( *Caryophyllia arnoldi* Vaughan, 1900 and *Crispatotrochus rubescens* (Moseley, 1880)) were included in the analyses as outgroups (see Table S1). Assembled reads were processed using the phyluce v 1.6.8 pipeline (Faircloth 2016), and UCEs and exon loci were bioinformatically extracted using the program ‘phyluce_assembly_match_contigs_to_probes’ that matched a combined scleractinian bait set (Cowman et al. 2020; Quek et al. 2020) to the assembled contigs with a minimum coverage and a minimum identity of 70%. Loci were then extracted into separate FASTA files using ‘phyluce_assembly_get_fastas_from_match_counts’ and aligned, using the edge trim, with default parameters using ‘phyluce_align_seqcap_align’ in MAFFT (Katoh et al. 2002). A data matrix of locus alignments was created in which each locus had at least 50% of species occupancy. A saturation test was run on the nuclear loci included in the data matrix (PhyloMAd; Duchêne et al. 2018) using entropy models on all sites, and all loci displaying substitution saturation were removed from further analyses. The remaining alignments were then concatenated, and the partition charset was created using ‘phyluce_align_format_nexus_files_for_raxml’. The concatenated alignment was used to perform a partitioned phylogenomic analysis using maximum likelihood (ML) in IQ‐TREE v 2.1 (Nguyen et al. 2015). The best‐fit models for each locus and best partition scheme were selected (−m MFP + MERGE) by ModelFinder (Kalyaanamoorthy et al. 2017) as implemented in IQ‐TREE v 2.1. Ultrafast bootstrap approximation (UFBoot) (‐B 1000; Hoang et al. 2018) was conducted as well as the SH‐like approximate likelihood ratio test (−alrt 1000; Anisimova et al. 2011).

The species tree analysis was conducted using ASTRAL III (Zhang et al. 2018). First, 378 gene trees (only alignments with 90% of taxon occupancy used—loci displaying substitution saturation excluded) were constructed using IQ‐TREE with the best‐fit models selected by ModelFinder. Trees were then concatenated into one file and input into ASTRAL III.

### 2.5 Mitochondrial Genome Analyses

Mitochondrial (mt) contigs were bioinformatically extracted using command line BLASTn (Altschul et al. 1990) against a set of reference scleractinian mt genomes. Annotation, using translation 4 (Mold, Protozoan, Coelenterate), and circularization were performed and manually verified using Geneious Prime 2024.0.5 (Biomatters Ltd. Auckland, New Zealand). The boundaries of all genes were then confirmed using BLAST against the NCBI nucleotide database. Base pair differences between complete mitogenomes were calculated using Geneious Prime 2025.1.2. Complete mt genomes retrieved in this study were also compared to an already published mt genome from the Mediterranean Sea (GenBank accession number: KC875349).

### 2.6 Morphological Characterization

Morphological observations of the colony and corallite of *D. pertusum* and *D. californicum* comb. nov. specimens were conducted with an Olympus SZX12 stereomicroscope (list of analyzed specimens in Taxonomic Account). Both specimens preserved in ethanol used for the phylogenetic reconstruction and dry specimens from the Smithsonian USNM collection were analyzed.

## 3 Results

### 3.1 Population Genomics

After filtering, 101 samples and 2496 unlinked SNPs were included in the *Desmophyllum* population genomic analyses. PCA identified three genetic clusters (Figure 2). The clusters included (1) Blake Plateau and Escarpment, east FL Slope, and Georges Bank Slope and Hudson Canyon sites, (2) Norfolk Canyon and the TX‐FL Slope, and (3) North Pacific. The first two PCs explained 7.7% and 5.8% of the observed variation. Similarly, based on the deltaK value, the STRUCTURE analysis indicated that three genetic clusters were likely (K = 3, Figure 3). There appeared to be admixture between all populations, but the Pacific samples were shown to have distinctly different genetic makeup from those in all other regions.

**FIGURE 2:**
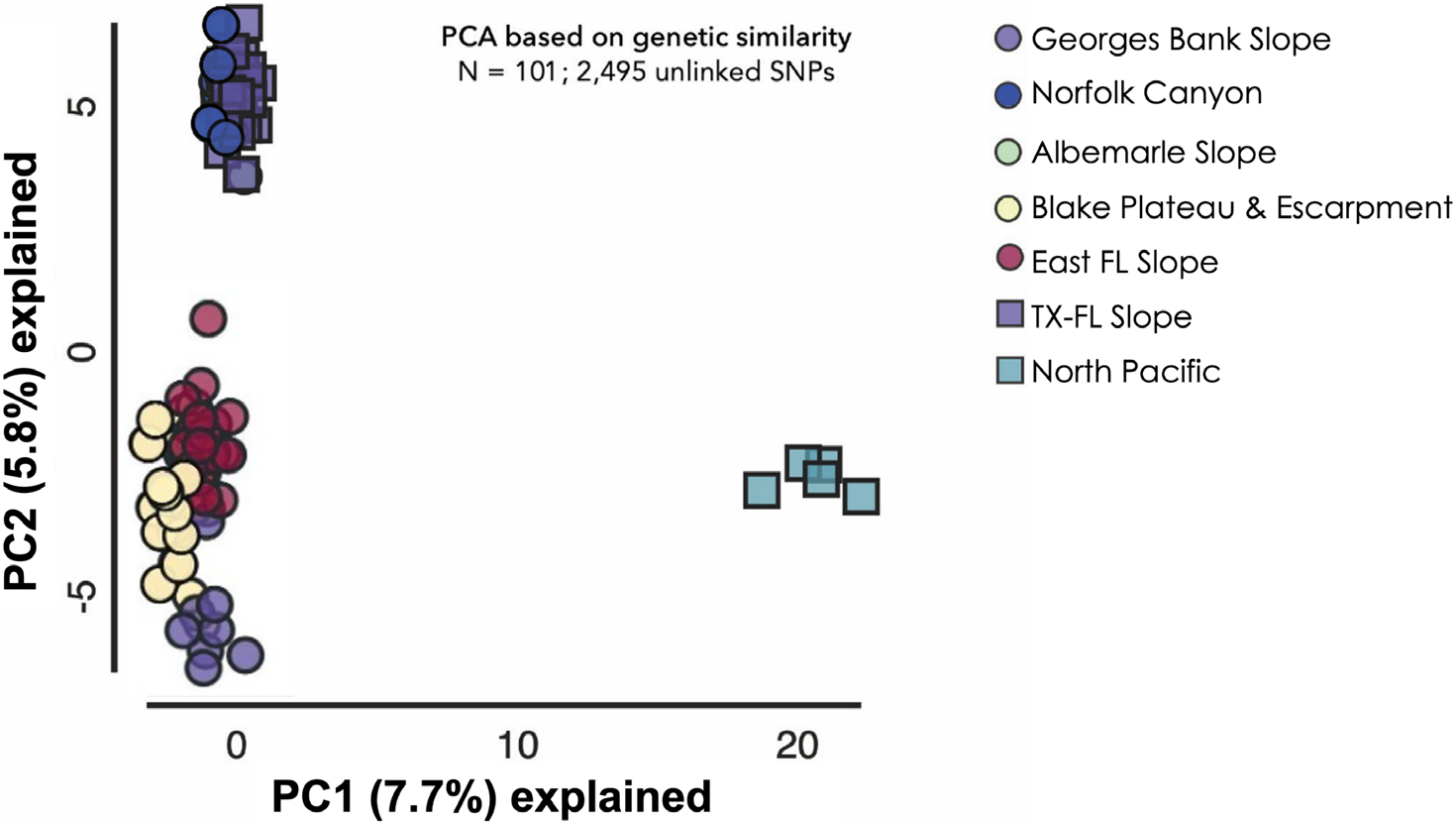
Principal component analysis (PCA) based on 2495 unlinked SNPs from 96 *Desmophyllum pertusum* and 5 *D. californicum* comb. nov. (North Pacific) individuals.

**FIGURE 3:**
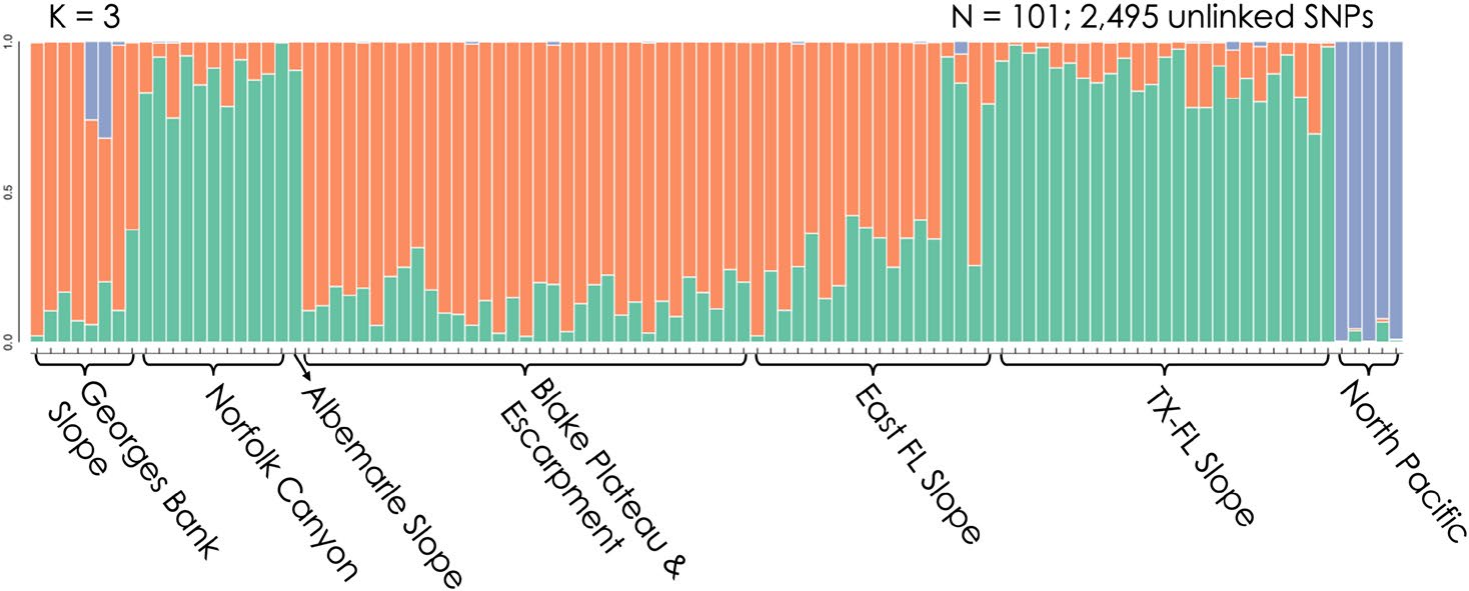
STRUCTURE analysis based on 2495 unlinked SNPs from 96 *Desmophyllum pertusum* and 5 *D. californicum* comb. nov. (North Pacific) individuals. *K* = 3 based on deltaK.

Genetic differentiation among sampling regions was also assessed using pairwise *F*_ST_ analysis, with values among the six regions ranging from 0.01–0.27 (Figure 4). The highest differentiation was observed between the North Pacific populations and all other regions, ranging from 0.23 (both the Blake Plateau and Escarpment population and the Georges Bank Slope population) to 0.27 (TX‐FL Slope population). In contrast, genetic differentiation was low (*F*_ST_ < 0.06) among populations from the Georges Bank Slope, Blake Plateau and Escarpment, and the east FL Slope. In addition, genetic differentiation was particularly low (*F*_ST_ < 0.02) between the TX‐FL Slope population and the Norfolk Canyon population, consistent with prior analyses (Weinnig et al. 2024). Thus, the magnitude and geographic pattern of pairwise *F*_ST_ estimates were concordant with the genetic clusters recovered by PCA and STRUCTURE, particularly the strong differentiation of the North Pacific population from Atlantic populations.

**FIGURE 4:**
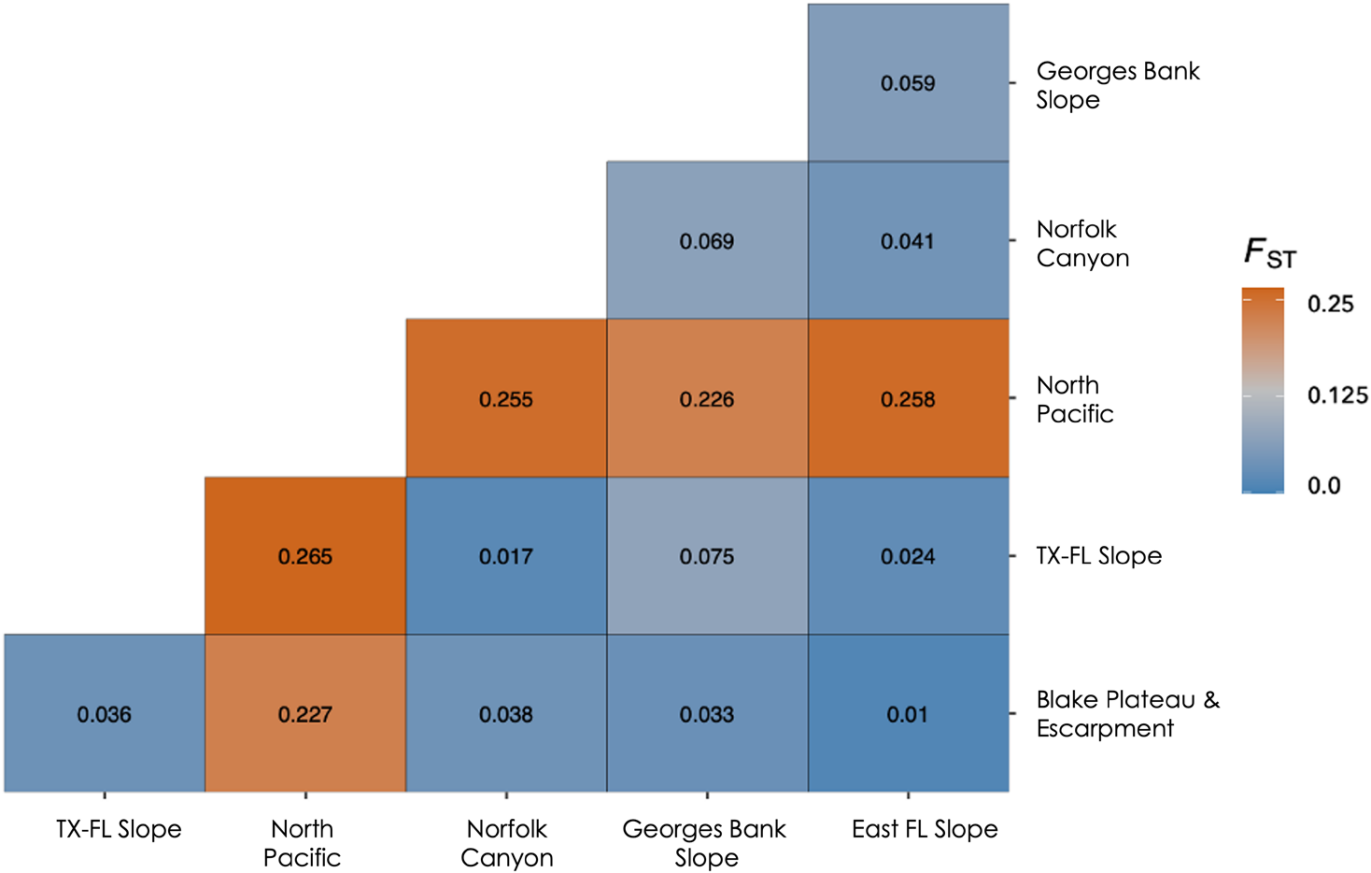
Pairwise FST values based on 2495 unlinked SNPs from 96 *Desmophyllum pertusum* and 5 *D. californicum* comb. nov. (North Pacific) individuals. Higher values are indicative of population isolation, and lower values indicate recent gene flow. TX = Texas, FL = Florida.

### 3.2 Nuclear Phylogenomics

For the nuclear dataset, a total of 2439 loci (out of 2490) across all 89 targeted specimens (83 sequenced for this study and six incorporated from previous studies—see Table S1) were recovered from the assembled contigs. The number of loci recovered from each specimen ranged from 397 to 1841 per sample. Only 14 loci displayed substitution saturation and were removed from further analyses. The final 50% taxon occupancy matrix included a concatenated alignment of 1794 UCEs and exon loci with an alignment length of 2,936,258 bp. The maximum likelihood and the species tree analysis resulted in identical (at deep nodes) and well‐supported topologies (Figures 5 and S1, respectively).

**FIGURE 5:**
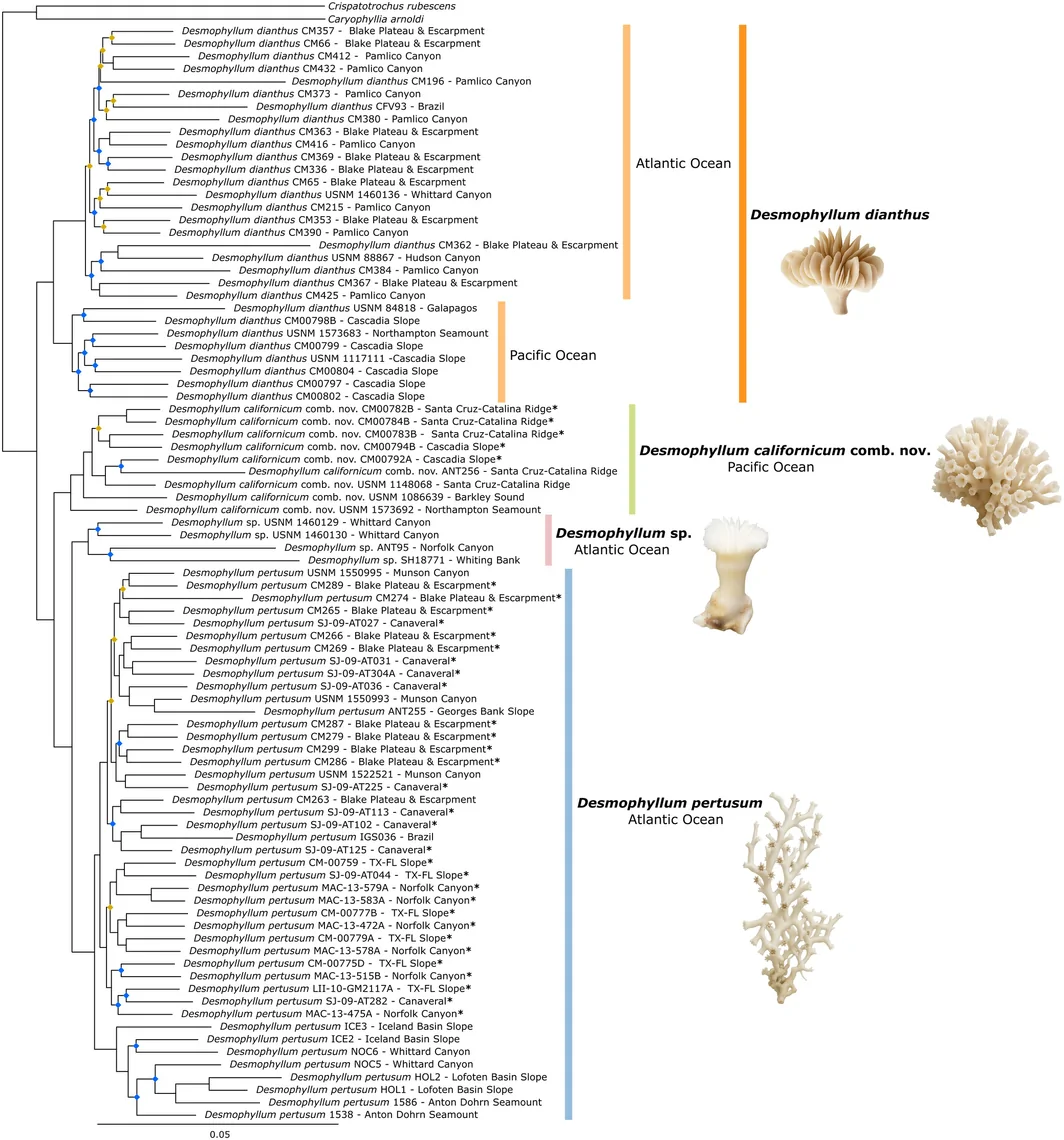
Maximum likelihood phylogeny based on the nuclear dataset (50% taxon occupancy—1794 UCEs and exon loci—2,936,258 bp). Nodes without diamonds indicate that SHaLRT and UFBoot are both 100. Blue diamonds indicate that both SHaLRT and UFBoot values are > 85; yellow diamonds indicate that either SHaLRT or UFBoot values are < 85. Colored bars indicate different species or lineages and correspond to those used in Figure 1. Asterisks indicate specimens that were used also for the population genomic analyses.

In the ML phylogeny (Figure 5), the majority of the *D. dianthus* specimens collected from the Atlantic Ocean were recovered as a monophyletic lineage (orange bars in Figure 5) (SH‐aLRT = 100, UFBoot = 100) sister to the *D. dianthus* collected in the Pacific Ocean (SH‐aLRT = 100, UFBoot = 100). These two lineages together were recovered as reciprocally monophyletic (SH‐aLRT = 100, UFBoot = 100) to a clade that includes: (i) *Desmophyllum pertusum* collected in the Atlantic Ocean (blue bar in Figure 5), (ii) initially identified *D. pertusum* collected in the Pacific Ocean (herein referred to *Desmophyllum californicum* comb. nov.) (green bar in Figure 5), and (iii) four specimens initially identified as *D. dianthus* and collected in the Atlantic Ocean (herein referred to as *Desmophyllum* sp.) (pink bar in Figure 5). Within this clade, *D. californicum* comb. nov. was retrieved as the most early diverging lineage, sister to *Desmophyllum* sp. and *D. pertusum* (SH‐aLRT = 100, UFBoot = 100). Notably, Atlantic *D. pertusum* specimens collected from the TX‐FL Slope and Norfolk Canyon grouped together in a clade sister to the remaining western north Atlantic Ocean *D. pertusum* specimens.

### 3.3 Mitochondrial Genomes

The complete mt genome was retrieved for seven specimens (four *Desmophyllum pertusum* and three *Desmophyllum californicum* comb. nov.—see Table 1 for details on selected specimens) sequenced for this study. All determined *Desmophyllum* spp. mt genomes are circular and range between 16,098 and 16,150 bp in size and from 34.8% to 34.9% in GC content. Numbers of base pair differences between the seven recovered mt genomes and a previously published *D. pertusum* mt genome (GenBank number KC875349—Flot et al. 2013) are specified in Table 1 and are found both in protein‐coding genes and intergenic regions. Two *D. pertusum* specimens (CM759 [Viosca Knoll] and MAC‐13‐579A [Norfolk Canyon]) have an identical deletion of 51 bp in the intron between the *nad5‐3′* and *cob* genes not present in any other retrieved mt for the species. All mt genomes present the three‐genes (*cob*‐*nad2*‐*nad6*) transposition already found in the genus *Desmophyllum* before (Emblem et al. 2011) and characteristics of the “true” Caryophylliidae lineage (Seiblitz et al. 2022).

**TABLE 1:** Number of mt genome base pair (bp) differences among specimens of *Desmophyllum californicum* comb. nov. and *Desmophyllum pertusum* from different localities (bp differences not accounting for the 51 bp deletion in the CM00759 and MAC‐13‐579A specimens). Voucher numbers and locations are given as in Figure 5. The GenBank number KC875349 represents a previously published mt genome (Flot et al. 2013).

		*Desmophyllum californicum* comb. nov.			*Desmophyllum pertusum*				
		USNM 1573692 (Northampton Seamount)	USNM 1086639 (Barkley Sound)	CM00792A (Cascadia Slope)	CM00759 (TX‐FL Slopel)	MAC‐13‐579A (Norfolk Canyon)	USNM 1522521 (Georges Bank Slope)	SJ‐09‐AT027 (East FL Slope)	KC875349 (Mediterranean Sea)
*Desmophyllum californicum* comb. nov.	USNM 1573692 (Northampton Seamount)	.							
	USNM 1086639 (Barkley Sound)	3	.						
	CM00792A (Cascadia Slope)	4	1	.					
*Desmophyllum pertusum*	CM00759 (TX‐FL Slope)	20	19	20	.				
	MAC‐13‐579A (Norfolk Canyon)	19	18	19	5	.			
	USNM 1522521 (Georges Bank Slope)	25	24	25	19	22	.		
	SJ‐09‐AT027 (East FL Slope)	34	33	34	28	29	13	.	
	KC875349 (Mediterranean Sea)	34	31	32	28	30	13	4	.

*Note:* Gray shading represents a heatmap of pairwise mt genome differences, with darker shades indicating a greater number of base pair differences.

### 3.4 Taxonomic Account

Order Scleractinia Bourne, 1900.

Suborder Vacatina Okubo, 2016.

Family Caryophylliidae Dana, 1846.

Genus *Desmophyllum* Ehrenberg, 1834.

***Diagnosis*** (amended from Addamo et al. 2016): Corallum solitary or colonial. Corallum of solitary species varies greatly in shape, trochoid with subcylindrical pedicel or cylindrical, ceratoid, or scolecoid. Colonial corallum is bushy in shape, often with anastomosing branches. Up to six cycles of septa non‐hexamerally arranged. Pali are absent. Columella is poorly developed or absent, visible only in early developmental stages. Septothecal wall in ontogenetically adult corallites is commonly covered by thickening deposits.

***Type species***: *Madrepora dianthus* Esper, 1794, pl. 69, fig. 1, 2, 3; by subsequent designation Cairns (1994, 76).

***Extant species included***: *Desmophyllum californicum* (Durham 1947); *Desmophyllum dianthus* (Esper, 1794); *Desmophyllum hourigani* Cairns, 2021; *Desmophyllum pertusum* (Linnaeus, 1758); *Desmophyllum quinarium* Tenison‐Woods, 1879.

***Desmophyllum pertusum*** (Linnaeus, 1758).

***Diagnosis***: See S. Cairns (2000).

***Distribution*:** Present throughout tropical and temperate waters in the Atlantic, Indian, and western Pacific Oceans; 40–2775 m.

***Remarks*:** *Desmophyllum pertusum* has long been considered a cosmopolitan species (see S. Cairns 2000); however, results from the present study clearly show that samples analyzed from the central and east Pacific Ocean correspond to a different species here resurrected (*Desmophyllum californicum* comb. nov.). It is possible that additional *Desmophyllum pertusum* populations from the west Pacific and Indian Oceans represent instead *D. californicum* comb. nov., thus further samples and studies could enhance understanding of the real distribution of *D. pertusum* .

***Desmophyllum californicum* (**Durham 1947 **) comb. nov., status revised**.

*Dendrosmilia nomlandi* Durham and Barnard, 1952:85, pl. 10: fig. 47—S. D. Cairns (1979):126– Bythell, 1986:16, pi. 10: fig. F; 1991a:18.

*Lophelia californica* Durham (1947):36, pl. 1: figs. 13, 16; pi. 2: fig. 11—Bythell, 1986:18, pl. 10: figs. A–E—Kozloff, 1987:72—S. D. Cairns (1991):17.

*Lophelia pertusa* S. D. Cairns (1994): in part (description and figs. 9e, i).

***Specimens examined***: USNM 19247, USNM 19250, Mexico—Baja California; USNM 1573692, Northampton Seamount, Hawaii; USNM 92606, USNM 1148068, Santa Cruz‐Catalina Ridge, California; USNM 1086639, Barkley Sound, British Columbia.

***Diagnosis*:** Corallum forms a bushy colony up to 25 cm in size, with anastomosing branches. Up to eight corallites can bud from the perimeter of a single corallite. Short cylindrical coenosteal bridges are present. Corallite diameter ranges from 4 to 6 mm, with some large corallites up to 11 mm in diameter. Corallum white. Septa irregularly arranged in three or four size classes. Columella is usually absent but occasionally expressed as a small crispate lath.

***Description*:** For a full description see S. D. Cairns (1994) (under *Lophelia pertusa* ). No new morphological observations are added in this study.

***Distribution*:** Pacific coast of North America, from Baja California to Canada, and Hawaii.

***Remarks***: *Desmophyllum californicum* comb. nov. was originally described as *Lophelia californica* by Durham (1947) from a single specimen collected by fishermen from “off coast of Southern California,” depth unknown. The description was brief, and the author did not include a comparison to other *Lophelia* species. The holotype was deposited at the Los Angeles County Museum (catalog number 2001) as of 2005. S. D. Cairns (1979) noted its resemblance to the Atlantic *Lophelia prolifera* (Pallas, 1766) (later synonymized to *L. pertusa* ) and stated that they were probably synonymous but did not formally include the species in his synonymy. Zibrowius (1980) considered *L. californica* to be the same as, if not identical to, *L. pertusa* but again did not synonymize the species, assuming that the distribution in different oceans might be sufficient ground for them being different species. In a paper about Galapagos fauna, S. D. Cairns (1991) finally synonymized *L. californica* with *L. prolifera* (= *L. pertusa* ) but without further comments. In a more comprehensive account, S. D. Cairns (1994) described and illustrated *L. californica* from northeastern Pacific specimens in his account of *Lophelia pertusa* . His specimens ranged from Cobb Seamount at ~46.8 N latitude (referenced in S. D. Cairns 1994 as “off Washington”) to Isla Guadalupe at ~28.8 N latitude (referenced in S. D. Cairns 1994 as “off Mexico”) at depths of 82–488 m. He also included a list of all east Pacific records of this species and names used for it. This should be considered to be the most recent and accurate description of *D. californicum* comb. nov.

Morphologically, *D. californicum* comb. nov. and *D. pertusum* differ primarily in budding (and thus colony shape) and corallite size. *Desmophyllum californicum* comb. nov. has closely spaced, intratentacular circumoral budding (rarely sympodial), with as many as eight corallites budding from the proximal edge of a single corallite (Figure 6d). This abundant branching leads to a bushy colony in which adjacent corallites often fuse together and sometimes are linked by short cylindrical coenosteal bridges (Figure 6b,c). The budding of *D. pertusum* is also intratentacular but primarily sympodial leading to a more elongate colony shape rather than being bushy (Figure 6a). Although corallites do sometimes fuse laterally, circumoral budding and coenosteal bridges are absent or very rare, and corallites are more widely spaced, leading to a more open corallum. Secondly, the corallites of *D. californicum* comb. nov. are smaller, usually 4–6 mm in greater calicular diameter (GCD), with occasional aberrant corallites up to 17 mm in GCD occurring in the same colony (Figure 6e) with otherwise small corallites. Corallites of *D. pertusum* are usually 10–15 mm in GCD, sometimes as large as 20 mm. Despite the size difference, both species have about the same number of septa arranged in a non‐hexameral pattern, i.e., 7–9 primary septa, 7–9 secondary septa, and 14–18 tertiary septa, for a total of 28–36 septa. Aberrant large corallites have correspondingly higher numbers of septa, up to 66 in some corallites. Otherwise, the two species are morphologically quite similar, as suggested by Cairns and Zibrowius (see above).

**FIGURE 6:**
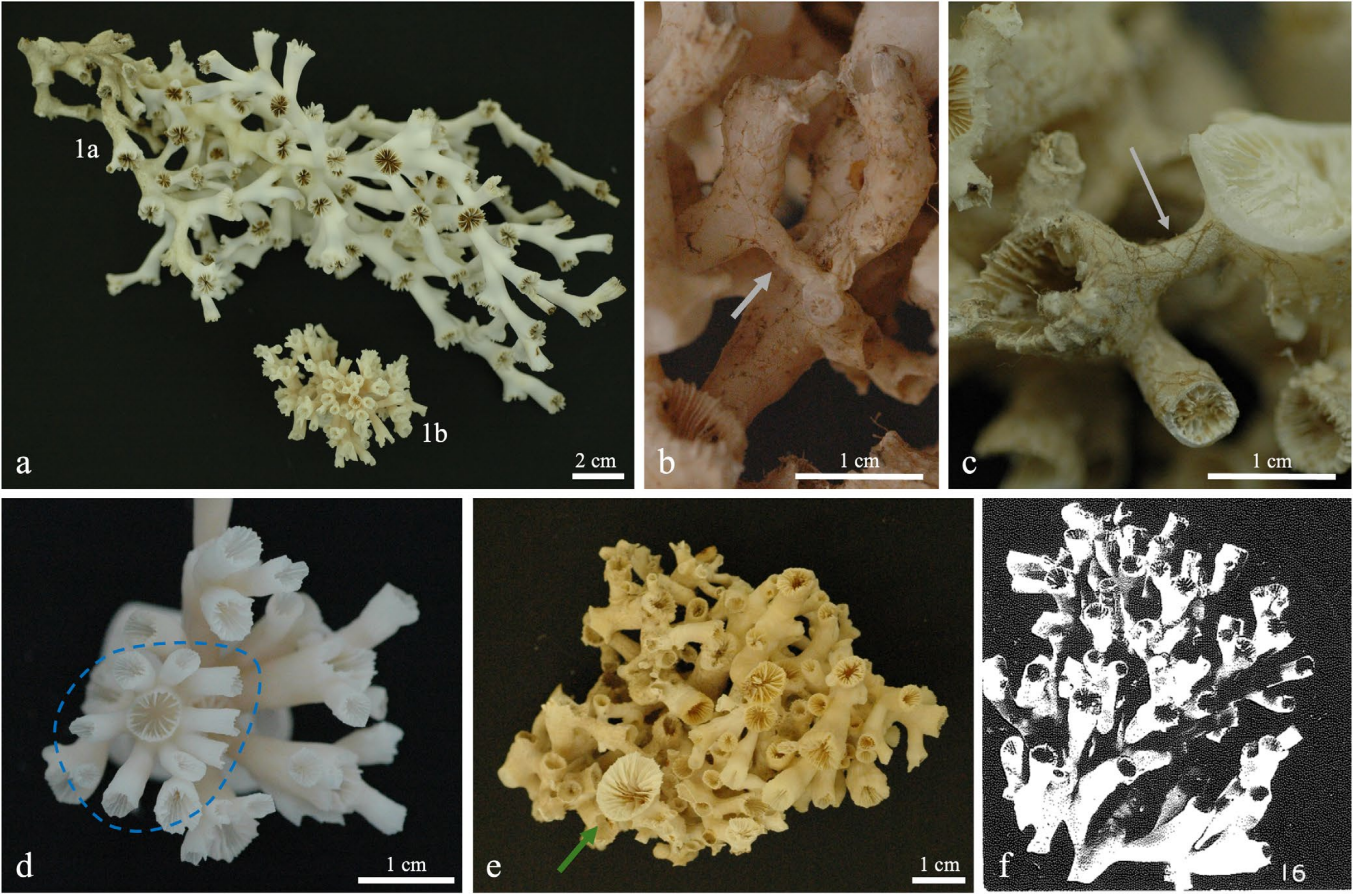
Museum specimens of *Desmophyllum pertusum* and *Desmophyllum californicum* comb. nov.; a image of *D. pertusum* USNM1437809 (1a) and *D. californicum* comb. nov. USNM92606 (1b) for colony and corallite size comparison; b‐c close‐up images of coenosteal bridges (gray arrows) in *D. californicum* comb. nov. specimens USNM19250 and USNM19247, respectively; image c also shows one aberrant large corallite on the right side of the arrow; d USNM92606, example of intratentacular circumoral budding in *D. californicum* comb. nov. with eight corallites budding from the proximal edge of a single corallite (dotted blue line); e USNM19250, *D. californicum* comb. nov. specimen displaying an aberrant large corallite (green arrow); f original image of the *Lophelia californica* holotype (LAM 2001, Los Angeles Museum Collection) from Plate 1 (partim) by Durham (1947).

## 4 Discussion

Our integrative approach supports the validity of the genus *Desmophyllum*, one of the most important coral genera that serves as a foundation species and ecosystem engineer in cold water and the deep sea. A decade before this study, Addamo et al. (2016) indicated that the genera *Lophelia* and *Desmophyllum—*with colonial and solitary traits, respectively*—*should be synonymized, and this became an enthusiastic discussion among cold‐water coral researchers. In our study, we present further evidence supporting the synonymy of *Lophelia* and *Desmophyllum*, based on phylogenomic data from 1000s of nuclear loci. Furthermore, species tree analyses indicate that solitary and colonial lineages are found in polyphyletic assemblages, and multiple genomic approaches (i.e., population genomics, mitogenomics, and phylogenetics) support several species in the genus.

### 4.1 Validity of the Genus *Desmophyllum*

Data based on nuclear UCEs and exons can reconstruct robust scleractinian phylogenetic relationships (e.g., McFadden et al. 2021; Quek et al. 2023; Vaga et al. 2024). Thus, for this study, we used both a maximum likelihood phylogenetic analysis based on a concatenated alignment and a species tree analysis that infers gene trees under a multispecies coalescent model. Both analyses supported a monophyletic *Desmophyllum* genus comprising at least two colonial and two solitary lineages, representing herein at least three valid species to date. Although coloniality—or the lack of it—has been historically used as a taxonomically informative character at the genus level (e.g., Cairns and Kitahara 2012), recent studies have shown that coloniality is a labile trait in the order Scleractinia (Campoy et al. 2020, Vaga et al. 2025) and not always reliable for taxonomic diagnosis (Addamo et al. 2016; Mehrotra et al. 2023). Finding high genetic and morphological similarity between *Lophelia* and *Desmophyllum* supported the idea of synonymizing these genera (Addamo et al. 2016). However, establishing monophyly is critical for taxonomy because monophyletic groups accurately reflect evolutionary history and relationships among species.

In the phylogenomic reconstruction, we found that a *Desmophyllum* sp. lineage (pink bar in Figure 5), containing specimens previously identified as *D. dianthus*, is genetically distant from all other *D. dianthus* . Based on our results, this lineage represents a new species for the genus or a previously described species that has been historically synonymized (at least nine junior synonyms, WoRMS) to *D. dianthus* based on morphological similarities (e.g., S. D. Cairns 1994, 1995). Given the small number of samples that could be analyzed in this study, it was not possible at the time to conclusively resolve the taxonomy of this lineage. Nevertheless, the two solitary *Desmophyllum* lineages are not monophyletic, nor are the two colonial *Desmophyllum* spp. (see next section below), which supports the validity of *Desmophyllum* as a genus.

### 4.2 *Desmophyllum californicum* Comb. Nov. Is a Valid Species

Many scleractinian species have been considered to have a cosmopolitan presence; however, studies have shown that morphological convergence and plasticity have often obscured the evolutionary distinctiveness of several lineages occurring in different ocean basins (Fukami et al. 2004; Arrigoni et al. 2016; Richards et al. 2016). In our results, both the maximum likelihood and species tree approaches recover the Pacific and Atlantic Ocean lineages of *D. pertusum* as clearly independent (i.e., polyphyletic—green and light blue bars in Figure 5) and genetically separated by a lineage comprising only solitary specimens (Figures 5 and S1).

Additionally, there is a clear distinction between the two species at a population level. The pairwise *F*_ST_, PCA, and STRUCTURE population genetic analyses consistently suggest that individuals from the Pacific Ocean represent a separate species rather than geographic population structure, particularly when considered together with the phylogenomic and morphological evidence. Although *F*_ST_ values are influenced by taxon‐specific and methodological factors and should not be interpreted as universal thresholds for species delimitation, values reported among independently delimited scleractinian lineages provide useful context for the magnitude of differentiation observed in this study. For example, interspecific or inter‐lineage *F*_ST_ values of 0.16–0.19 have been reported in *Favia* (Adam et al. 2024), while values spanning 0.19–0.52 and 0.266–0.616 have been reported among distinct genomic taxa or species‐level clusters of *Stylophora* and *Pocillopora*, respectively (Meziere et al. 2024; Connelly et al. 2026). The individuals from the North Atlantic Ocean are likely all *Desmophyllum pertusum*, and even though there are multiple distinct populations, further studies could confirm this. These results mirror Weinnig et al. (2024) findings, which show a strong population differentiation between the TX‐FL Slope and Norfolk Canyon populations relative to the Blake Plateau and Escarpment and the Georges Bank Slope and Hudson Canyon populations. The pairwise *F*_ST_ values are consistent with a previous analysis of a subset of these samples (Weinnig et al. 2024). Altogether, results indicate that there is high connectivity and genetic similarity among *D. pertusum* populations along the east Florida Slope, Blake Plateau and Escarpment, and Georges Bank Slope and Hudson Canyon. The addition of *Desmophyllum californicum* comb. nov. individuals to the population‐level analysis supports the finding that there is strong differentiation between populations in the two basins and that the individuals from the Pacific Ocean are not only a distinct population but comprise a different species.

*Desmophyllum pertusum* is a frequently collected and thus well‐known species; comprehensive descriptions of the species can be found in both S. D. Cairns (1979) and Zibrowius (1980). It is quite a variable species morphologically, which led earlier authors to synonymize *L. californica* with *L. pertusa* . Their variation was considered to be environmental or population level. But with compelling molecular evidence herein to support the distinction of these two species, we were led to re‐evaluate the morphological differences and conclude that they in fact could be used to distinguish between two separate species: *Desmophyllum pertusum* and *Desmophyllum californicum* comb. nov.

### 4.3 Intraspecific Phylogenetic Relationships

Both ML and species tree reconstruction (Figures 5 and S1) recovered *D. pertusum* from the Atlantic Ocean as a monophyletic lineage. However, inside this lineage, some phylogroups at the population level can be observed, as found by Weinnig et al. (2024). Specimens from the northeast Atlantic Ocean form a sister clade to those from the western Atlantic Ocean, consistent with a pattern of isolation by distance (e.g., Polato et al. 2010; Ballesteros‐Contreras et al. 2022). The majority of the populations from the east Florida Slope north to the Georges Bank Slope form an admixed lineage, with the exception of specimens from the Norfolk Canyon, which appear to be more closely related and intermingled with specimens from the continental slope between Texas and Florida.

*Desmophyllum dianthus* specimens collected in the Atlantic and Pacific Oceans are respectively forming separate, reciprocally monophyletic, and well‐supported lineages (Figures 5 and S1). This result might suggest the presence of strong population structure between the two basins or, alternatively, that one lineage represents an additional new species. Because the gross morphology of these two groups is indistinguishable, population genomic analyses, a greater number of samples, additional ecological data, and more detailed quantitative morphological analyses could provide further information on the taxonomy and systematics of these *D. dianthus* lineages.

### 4.4 Mitochondrial Genomes

The mt genomes from both *D. pertusum* and *D. californicum* comb. nov. have length and GC content that match other *Desmophyllum* specimens (Emblem et al. 2011; Addamo et al. 2016; Seiblitz et al. 2022). Complete mt genome data for multiple specimens of the same species are seldom available for Scleractinia. However, a previous study (Seiblitz et al. 2022) retrieved a maximum of 15 bp differences among mt genomes of Atlantic *D. pertusum* specimens. *Desmophyllum californicum* comb. nov. specimens show almost no mt differences among them (≤ 4 bp), even between geographically distant locations (i.e., Hawaii and Pacific coast of the continental U.S.), but they have up to 34 bp differences (99.79% of identity) when compared to the Atlantic *D. pertusum* (Table 1). This percentage of identity is lower than the one found (99.88% similarity) between the two clearly separate species *D. dianthus* and *D. pertusum* (Addamo et al. 2016). This result further corroborates the separation between the two colonial *Desmophyllum* species. Interestingly, specimens of the slope from Texas to Florida and the Norfolk Canyon lineage also show high mt genetic diversity when compared to the rest of the Atlantic specimens (Table 1). Moreover, their mt genome has a 51 bp deletion not present in other specimens. These results, once more, reinforce the previous findings about genetic discontinuity among the *D. pertusum* population in the northwestern Atlantic Ocean (Weinnig et al. 2024).

### 4.5 Conservation Implications of Taxonomic Revisions

Given its ecological significance and the wealth of research and conservation efforts surrounding *D. pertusum* over the past few decades, the suggestion that Pacific Ocean populations represent a different species is far from inconsequential. This is particularly meaningful in light of the recent inclusion of the Atlantic *D. pertusum* in the International Union for Conservation of Nature (IUCN) Red List of Threatened Species as Vulnerable (Sigwart et al. 2025). Most of the research and conservation efforts to date have occurred under the name *Lophelia pertusa* , and changes to the nomenclature are often met with resistance and are slow to gain acceptance by the scientific community. The analyses presented here strongly support the synonymy of *Lophelia* with *Desmophyllum*. Because *Lophelia* remains the term most widely recognized across scientific databases, management agencies, and international conservation programs, a concerted effort to update these usages will be required. Changing the genus from *Lophelia* to *Desmophyllum* may introduce challenges in management and conservation, especially in the north Atlantic Ocean, such as the need to edit the text of legislation protecting the species. Establishing the consistent use of the correct scientific name is important to ensure continuity across scientific, management, and conservation communities, and efforts to communicate the most current taxonomic understanding may help to maintain a clear link to decades of prior research.

Understanding the full diversity and distribution of *Desmophyllum* species could be further enhanced. For example, there is likely at least one new species of solitary *Desmophyllum*. Additionally, *D. californicum* comb. nov. probably occurs in other Pacific localities not included in this study. Therefore, further analyses could clarify the true geographic distribution of *D. californicum* comb. nov. and identify more cryptic species. Our integrative taxonomic approach, supported by genomic data, suggests that multiple *Desmophyllum* lineages represent valid species beyond those currently recognized.

## Author Contributions

**Claudia F. Vaga:** conceptualization (lead), data curation (lead), formal analysis (lead), investigation (lead), methodology (lead), resources (supporting), supervision (lead), validation (lead), visualization (lead), writing – original draft (lead), writing – review and editing (lead). **Alexis M. Weinnig:** conceptualization (lead), data curation (lead), formal analysis (lead), funding acquisition (supporting), investigation (lead), methodology (lead), resources (supporting), supervision (lead), validation (lead), visualization (lead), writing – original draft (lead), writing – review and editing (lead). **Stephen D. Cairns:** investigation (supporting), supervision (supporting), writing – original draft (supporting), writing – review and editing (supporting). **Michelle L. Taylor:** investigation (supporting), resources (equal), validation (supporting), writing – review and editing (supporting). **Meredith Everett:** resources (supporting), writing – review and editing (supporting). **Santiago Herrera:** resources (supporting), writing – review and editing (supporting). **Erik E. Cordes:** resources (supporting), writing – review and editing (supporting). **Andrea M. Quattrini:** conceptualization (equal), funding acquisition (lead), investigation (equal), methodology (equal), project administration (equal), resources (lead), supervision (lead), writing – review and editing (equal). **Cheryl L. Morrison:** conceptualization (equal), funding acquisition (lead), investigation (equal), project administration (equal), resources (lead), supervision (equal), validation (equal), visualization (equal), writing – review and editing (equal).

## Funding

This work was supported by Bureau of Ocean Energy Management, contract M08PC20038, contract M10PC0010, contract M17PC00009. Coral Endowment Fund at the Smithsonian Institution. Mesophotic and Deep Benthic Communities Habitat Assessment and Evaluation Project.

## Conflicts of Interest

The authors declare no conflicts of interest.

## Supporting information


**Figure S1:** Species tree. ASTRAL III species tree with posterior probabilities calculated in ASTRAL.

**Table S1:** Information and details about voucher number, collection location, depth, and analyses undertaken for each sample included in the manuscript.

## Data Availability

Complete mitochondrial genomes were deposited in the GenBank repository (accession numbers PZ269946‐PZ269952). Raw read data were uploaded to the Sequence Read Archive under the BioProject PRJNA1449395—under the umbrella Mesophotic and Deep Benthic Community restoration project PRJNA1135238. BioSample numbers SAMN57124159‐SAMN57124231. SRA numbers SRR37979276‐SRR37979283, SRR37983193‐SRR37983202, SRR38000977‐SRR38001018, SRR38023100‐SRR38023120. Already published and deposited in GenBank sequences used to build the alignments can be found in the BioProjects PRJNA1027916 and PRJNA1261827.
