# Supplementary material for: Integrative Taxonomy Does Not Support a Cosmopolitan Desmophyllum pertusum: Resurrecting D. californicum and Validating the Genus Desmophyllum
Source: Ecol Evol. 2026 Oct 16;16(10):e74485. doi: 10.1002/ece3.74485 (PMC13674452; doi:10.1002/ece3.74485)

Title: Integrative taxonomy does not support a cosmopolitan *Desmophyllum pertusum*: Resurrecting *D. californicum* and validating the genus *Desmophyllum*

Authors: Claudia F. Vaga, Alexis M. Weinnig, Stephen D. Cairns, Michelle L. Taylor, Meredith Everett, Santiago Herrera, Erik E. Cordes, Andrea M. Quattrini, Cheryl L. Morrison

Disclaimer: Any use of trade, firm, or product names is for descriptive purposes only and does not imply endorsement by the U.S. Government.

Supplementary Figure 1| Species tree. ASTRAL III (Zhang et al. 2018) species tree with posterior probabilities calculated in ASTRAL.

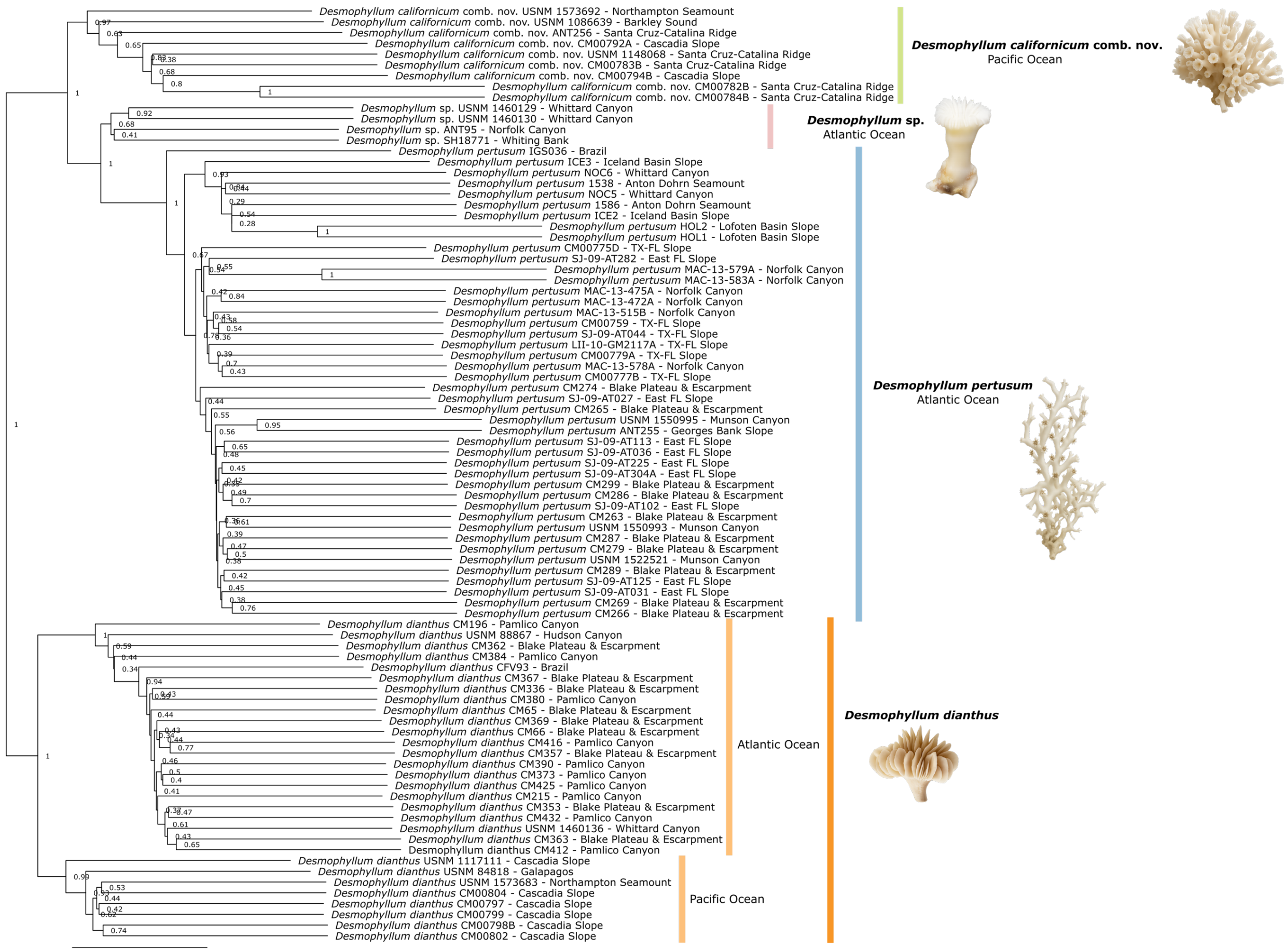

Supplement: Supplementary file 1 [file ECE3-16-e74485-s001.pdf]
